# Effect of Hybrid Nanofluids Concentration and Swirling Flow on Jet Impingement Cooling

**DOI:** 10.3390/nano12193258

**Published:** 2022-09-20

**Authors:** Ooi Jen Wai, Prem Gunnasegaran, Hasril Hasini

**Affiliations:** 1 Institute of Power Engineering, Putrajaya Campus, Universiti Tenaga Nasional, Jalan IKRAM-UNITEN, Kajang 43000, Malaysia; 2 Department of Mechanical Engineering, College of Engineering, Putrajaya Campus, Universiti Tenaga Nasional, Jalan IKRAM-UNITEN, Kajang 43000, Malaysia

**Keywords:** hybrid nanofluid, jet impingement, heat transfer enhancement, swirl flow

## Abstract

Nanofluids have become increasingly salient in heat transfer applications due to their promising properties that can be tailored to meet specific needs. The use of nanofluids in jet impingement flows has piqued the interest of numerous researchers owing to the significant heat transfer enhancement, which is vital in the technological dependence era in every aspect of life, particularly in engineering applications and industry. The aim of this current work is to investigate the effect of hybrid nanofluids concentration and swirling flow on jet impingement cooling through experimental approach. The hybrid nanofluids are prepared through a two-step method and the characterization process is carried out to study the stability and morphological structure of the sample prepared. The prepared hybrid nanofluids are then used as a cooling agent to evaluate the heat transfer performance of jet impinging system. The experimental investigation compares the performance of swirling impinging jets (SIJs) with conventional impinging jets (CIJs) under various jet-to-plate distance (H/D) ratios and nanofluid concentrations. The effects of adding surfactant on nanofluids are also examined. The heat transfer performance of ZnO/water and CuO/water mono-nanofluids are used as comparison to ZnO-CuO/water hybrid nanofluid. The results show that the thermal performance of ZnO-CuO/water hybrid nanofluid is better than that of the mono-nanofluids. Furthermore, as the mass fraction increases, the heat transfer rates improve. The effect of heat transmission by swirling impinging jets is better than that of conventional impinging jets under similar operating conditions. At H/D = 4, Re = 20,000 and hybrid nanofluid concentration at 0.1% under SIJ is observed to have the highest overall Nusselt number.

## 1. Introduction

The liquid cooling system that uses single phase heat transfer fluids such as engine oil, propylene glycol, ethylene glycol, transformer oil, and water is largely used in thermal and chemical power plants. In the modern era, single phase heat transfer fluid has limited performance as it has a very low thermal conductivity; thus, it is not sustainable in the current market demands for better performance, longer lifespan, and size reduction. Therefore, the idea came to improve the thermal conductivity of the working fluids by introducing solid particles into the fluids as solid materials by nature possess higher thermal conductivity. This innovative idea was first proposed by Maxwell [1] in 1873, where he observed enhanced thermal conductivity but raised multiple issues such as sedimentation which causes clogging on the flow passage wall that eventually leads to erosion of the pipes and increased pressure drop in the system. Years later, in 1993, Masuda et al. [2] attempted a similar technique using dispersed micro-meter size solid particles into the single-phase fluids but encountered similar results where there was particle sedimentation in the base fluid. The most significant improvement in the practical applications of heat transfer fluid was observed in 1995 when Choi [3] introduced nanoscale carbon nanotubes and metallic particles. The dispersion of nanoscale particles in base fluids display higher specific surface area and higher stability when compared to fluids containing the micro and milli-sized solid particles. With this development that revolutionized heat transfer fluid, researchers have been attracted to exploring the hidden potential of nano-fluids [4].

Over the last couple decades, research into nanotechnology has rapidly grown due to the combination of thermal engineering that led to the new heat transfer fluids termed “nanofluids”. A nanofluid is a heat transfer fluid that contains suspended nanometer-sized particles known as “nanoparticles”, which are in the range of 1–100 nm, dispersed in the base fluid. It is very important to ensure the size of the nanoparticles is less than 100 nm to achieve a higher stability of the working fluid. There are typically 5 types of nanoparticles used to produce nanofluids, namely metals such as gold (Au), silver (Ag), nickel (Ni), and copper (Cu); metal carbides such as silicon carbide (SiC); metal oxides such as zirconium oxide(ZrO_2_), iron (II) oxide (Fe_3_O_4_), iron (III) oxide (Fe_2_O_3_), aluminum oxide (Al_2_O_3_), silicon dioxide (SiO_2_), copper oxide (CuO), zinc oxide (ZnO), and titanium dioxide (TiO_2_); carbon materials such as carbon nanotubes, graphene, and diamond; or metal nitrides such as aluminum nitrite (AlN) and boron nitride (BN) [5,6]. The present of nanoparticles improves the heat transfer coefficient and performance of the base fluids tremendously. The base fluids that are commonly used are water, oils, organic liquids (e.g., refrigerants, tri-ethylene-glycols, ethylene, etc.) or bio-fluids polymeric solution [7,8].

Throughout the years, various research has reported a variety of nanofluid preparations with different types of nanoparticles and its heat transfer performance along with the development of nanofluids’ relevant knowledge. Strengths and weaknesses of these nanofluids have been recorded and compared by measuring their hydrodynamic and thermophysical properties. An experiment conducted with TiO_2_, Al_2_O_3_, and Cu nanofluids with water as base fluids reveled that Cu nanofluids provided comparatively better results with higher overall thermal performance [9]. Researchers have also delved into the possibilities of predicting the thermal properties of nanofluids using Gaussian process regression (GPR) model, where the thermal conductivity enhancement percentage and specific heat capacities of the nanofluids are able to be predicted across various ranges of composition and temperatures tested [10,11].

Over the years, nanofluids demonstrated satisfactory results to the point where researchers started to ponder on the combinations of different nanoparticles dispersed in base fluid, which were later developed and referred as “hybrid nanofluids”. A hybrid material is a substance which has the capability to combine both chemical and physical properties of two or more different materials simultaneously at nanometer or molecular level and provides these properties in a homogeneous phase. Obtaining the properties of its constituent materials is the main reason behind fabricating the hybrid nanofluid, as a single material does not hold all the desirable characteristics to function well for a specific purpose. For instance, it may either have good rheological properties or thermal properties. In most engineering applications, it is necessary for the materials selected to trade off between several properties and that is where the adoption of hybrid nanofluid becomes relevant. This rising class of nanofluids demonstrated significant improvement in terms of hydrodynamic properties, thermophysical properties, and heat transfer characteristics when compared to individual nanofluids due to synergistic effect [12].

Sun et al. [13] applied a hybrid nanofluid containing the Ag-multiwall carbon nanotube nanoparticles at 50:50 ratio in a jet impinging cooling system. The outcomes showed that the thermal conductivity of Ag-MWCNT/water hybrid nanofluid enhances significantly when compared to MWCNT/water nanofluid. An investigation of Ag-CuO/water hybrid nanofluid in rotating three-dimensional steady flow revealed that it has better heat transfer performance when compared to unitary nanofluid even under the presence of thermal radiation, chemical reaction, and heat generation [14]. Hybrid single-walled nanotube with metal oxide such as switched walled carbon nanotubes (SWCNTs)/MgO-ethylene glycol hybrid showed enhanced thermal conductivity compared to single-particle nanofluids [15]. Heat transfer behavior and rheological characteristics of hybrid nanofluids were further studied with copper-titanium dioxide (Cu-TiO_2_) nanocomposite that was dispersed in water as base fluid [16]. The experiment was conducted with different particle concentrations ranging from 0.1 to 1 (mass fraction %) in base fluid. It was observed that the Nusselt number exhibited an increasing trend until 0.7% particle concentration and further increase in concentration resulted in a decline of the Nusselt number. This scenario occurred because of the agglomeration (cluster formation) as gap between particles are reduced due to increased Van der Waals forces within a higher particle concentration and thus increases the chances of cluster formation [17]. Different nanoparticles may experience different mass fraction concentration as each particle has different Van der Waals forces of attraction. Common nanofluid preparation methods such as one-step and two-step methods are used for the production of hybrid-based nanofluids.

The liquid working fluid in jet impingement systems is usually water or ethylene glycol. As technologies advances, the traditional working fluids no longer could meet the required demands to dissipate heat. Hence, due to the rise of nanofluid research, it has prompted researchers to merge this state-of-the-art working fluid to jet impingement cooling to replace the conventional working fluid. Such implementation resulted in positive effects where the heat transfer performance improved remarkably, reducing the weight and size of the jet impingement design, which led to lower capital cost in fabricating the cooling system [16,18]. Heat transfer enhancement in jet impingement cooling with CuO/water nanofluid was experimentally performed by Modak et al. [19], who concluded with three reasons why the presence of nanofluid in jet impingement improved the heat transfer rate. First of all, the suspended nanoparticle in base fluid enhanced the thermal conductivity of the working fluid. Next, there was the bombardment of the nanoparticles on the target surface after the nanofluid emerged from the exit nozzle. This process resulted in higher turbulence and heat transfer rate while reducing the boundary layer thickness. Lastly, wettability enhancement on the targeted surface was stated as the third reason when a thin layer of nanoparticles deposited was observed using SEM. From the experiment, they obtained a contact angle of 54.3° using nanofluids compared to 85.8° using a water-impinged surface. The lower contact angle indicated enhanced wettability, which means the nanofluid droplet spread out more over the surface when compared to water, thus higher heat transfer rate occurred.

The jet configurations can be classified into five categories which is free surface, submerged, confined, wall, and plunging jet impingement. Free surface jet is established when the liquid working fluid is ejected into the ambient gas while the submerge jet occurs when the working fluid is ejected into the same liquid medium. From the past reported journals [20,21,22,23,24], the studies of potential core are more relevant in the submerged jet configuration due to the effect of the surrounding medium upon exiting the jet nozzle, as entrainment of surrounding fluid in the free surface configuration into the gas medium can be considered negligible. A clear distinction was observed between the free surface jet and submerged jet, especially in the effect of entrainment and interaction modes. Further examination of the submerged jet impingement system reveals that it can be divided into two categories, confined and unconfined jet. The working fluid is entrained and recirculated back into the impinging jet in the confined jet impingement, resulting in the formation of recirculation zones at the outlet flow regions. This, however, does not occur in the unconfined jet as the heated working fluid is not returned into the impinging jet after the jet interaction with the passive ambient surroundings. Therefore, the unconfined jet tends to have higher heat transfer coefficients when compared to confined jet. Both confined and unconfined jet are often used as cooling solutions as each have their own merits. Confined impinging jets are usually chosen when limited space is provided as it has a small space design. On the other hand, unconfined jets have the advantages of easy fabrication and simplicity in design when compared to confined jets [25].

**Figure 1:**
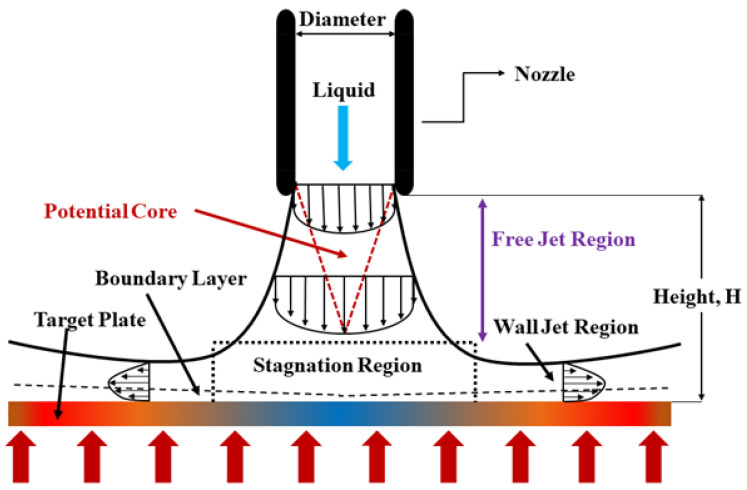
Flow structure of free surface jet.

The flow structure of a conventional liquids jet before and after striking the target surface often experience three distinct regions, which are free jet, stagnation, and wall jet region as represented in Figure 1. The free jet region is the area where the working fluid is ejected and diverged from the jet nozzle. The length of this region is dependent on various parameters, such as the jet shape, jet to target spacing, and nozzle exit conditions. Three common phases make up this region, which is the developing flow, fully developed flow, and potential core. As for the developing phase, the centerline velocity decays while the fully developed phase has a similar velocity profile. On top of that, the velocity in the potential core is constant and equal to the exit jet velocity. When the working fluid strikes the target plate, the stagnation region is formed; the length of this region ends when zero pressure gradients is achieved on the target plate. The characteristic of the flow at this region often has a very high strain and strong curvature because of the existence of an impingement wall. The axial velocity drops drastically while the static pressure and radial velocity increase in this region. Lastly, the transverse flow velocity of the working fluid at the wall jet region is depleted as the distance it travelled increases. Higher heat transfer rate occurs at this region due to higher turbulence intensity formed due to the shear force created between the liquid and impinged wall [26].

Research on the hybrid nanofluids have been increased very rapidly over the past decades. In spite of some inconsistency in the reported results and insufficient understanding of the mechanism of the heat transfer in hybrid nanofluids, they have emerged as a promising heat transfer fluid. Hybrid nanofluids offer exciting new possibilities to enhance heat transfer performance compared to conventional fluids. Although there have been many publications on hybrid nanofluids, there is not sufficient research over the years that focuses on its use in jet impingement systems. As a result, the full potential of hybrid nanofluids in jet impingement is yet to be fully discovered. Hence, this paper reduces the research gap by extending the contribution of heat transfer enhancement knowledge through experimenting with novel ZnO-CuO/water hybrid nanofluid as the working fluid in a jet impingement cooling system. The present work provides a potential heat dissipation solution in conjunction with the rise in demand for high performance electronic devices and parts, as well as their miniaturization. The aim of this research is to study the thermophysical and hydrodynamic properties of hybrid nanofluid and the effect of geometrical parameters of jet impingement cooling using hybrid nanofluid as working fluid.

## 2. Materials and Methods

### 2.1. Hybrid Nanofluid Preparation

In this experiment, hybrid nanofluids are prepared by the two-step method with ZnO and CuO nanoparticles. The basic thermophysical properties of both the nanoparticles are shown in Table 1. The two-step method involves dispersing nanoparticles in base fluid, which in this study is deionized water. Production of nanofluid using this method is widely used on a large scale as it is more economical and simpler [27]. The main drawback of this method is the agglomeration due to the van der Waals and cohesive strength of the independent nanoparticles. There are several ways to suppress the agglomeration, such as utilizing dispersants or surfactants at appropriate concentrations or continuously allowing the working fluid to undergo ultrasonic baths after jet impinging cooling [28].

**Figure 2:**
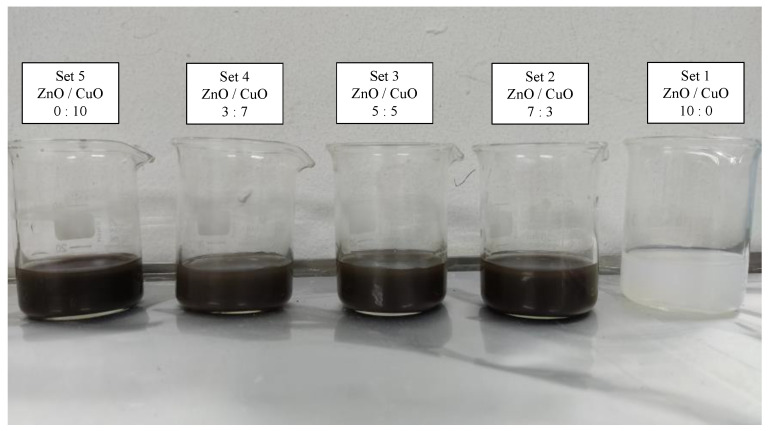
Samples of prepared hybrid nanofluids.

For this experiment, to ensure longer stability and complete dispersant of nanoparticles into the base fluids. The samples were blended with 3 h of magnetic stirrer and followed by 6 h of ultrasonic bath both at a constant temperature of 50 °C. Stability of the prepared nanofluid was enhanced through this method of preparation. Five different ratios of mixed solution of ZnO and CuO were prepared at 10:0, 7:3, 5:5, 3:7, and 0:10. Each of the nanoparticle weights was measured using an analytical balance before it was mixed in a 2 liter beaker. Each of the five ratio samples was prepared at 0.1% mass fraction and underwent material characterization as shown in Figure 2. Considering the thermophysical properties of the hybrid nanofluids, the ratio of 5:5 was selected for the jet impinging cooling study. The presence of two different particles effectively increased the specific surface area, which led to higher collisions rate between the particles, which enhanced the heat transfer. Hybrid nanofluid of the ZnO-CuO/water nanofluid and mono-nanofluid of ZnO and CuO were prepared at mass fractions of 0.01%, 0.025%, 0.05%, 0.075%, and 0.1% to be used as cooling fluids for the investigation of jet impingement cooling. Another set of similar mass fraction of hybrid nanofluids was prepared with added sodium dodecylbenzene sulfonate (SDBS), a surfactant, to study the effect on hybrid nanofluids stability.

### 2.2. Characterization of Hybrid Nanofluid

The characterization of the hybrid nanofluid was determined using six different methods. These included scanning electron microscopy (SEM) to study the surface morphology and topology of the hybrid nanofluid. The SEM was also equipped with the Energy Dispersive X-ray Analysis (EDX) option whereby the element distribution of the region of interest could be identified. The hybrid nanofluid stability was evaluated by using visual observation to observe the sedimentation of the hybrid nanofluid several days after preparation. For more accurate results, the zeta potential test was used to obtain the stability agglomeration of the hybrid nanofluid by measuring its absorbance property [1,2]. The thermal conductivity of the hybrid nanofluid was analyzed using the KD2 probe to determine its thermal conductivity. As for the dynamic viscosity of the hybrid nanofluid, a rheometer that is capable of measuring temperature range of 20–80°C was used for characterization study.

#### 2.2.1. Scanning Electron Microscope (SEM)

**Figure 3:**
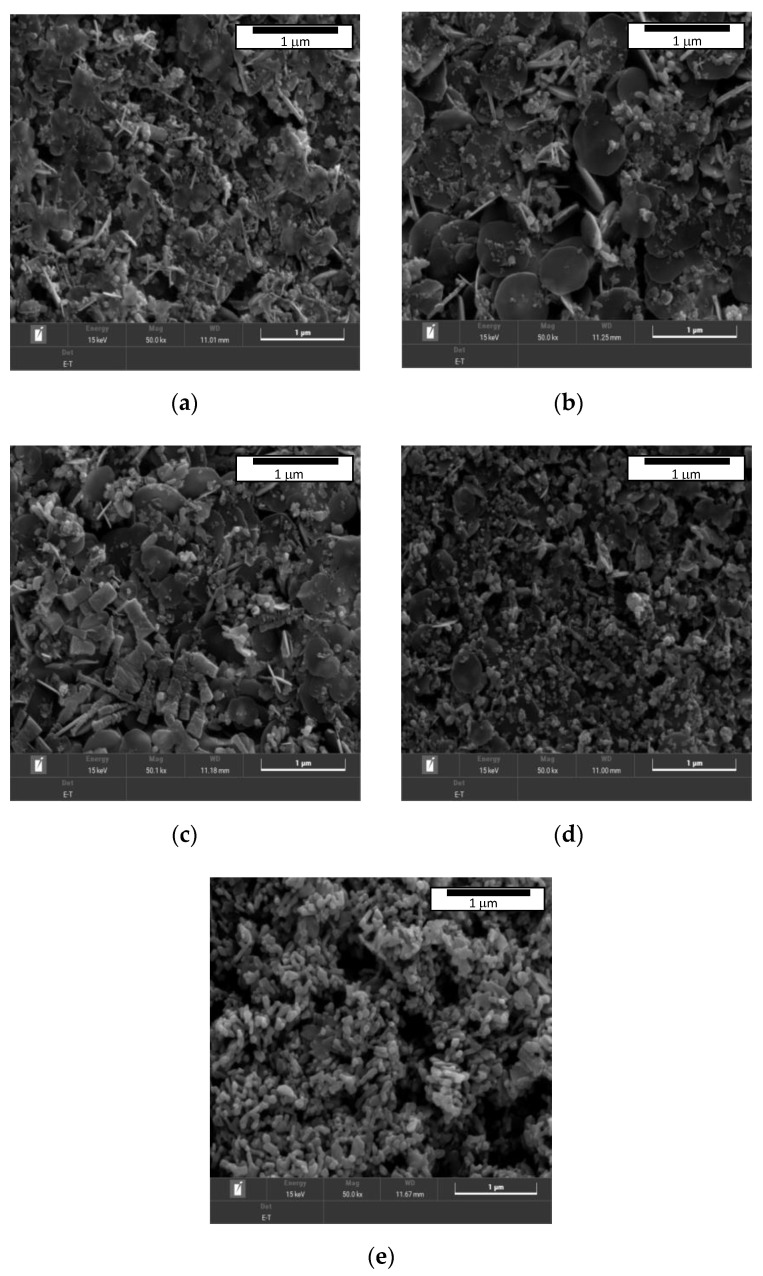
SEM image of ZnO-CuO/water hybrid nanofluid with magnifications of 50 k× mixed at ratio of (**a**) 10:0, (**b**) 7:3, (**c**) 5:5, (**d**) 3:7, (**e**) 0:10.

To further understand the microstructure and morphological structure of the hybrid nanofluid at different ratio, it was observed under scanning electron microscope (SEM). The SEM images of the ZnO-CuO/water hybrid nanofluid mixed at five different ratios, 10:0, 7:3, 5:5, 3:7, and 0:10, are shown in Figure 3. All samples of the hybrid nanofluids characterized under SEM were prepared through two step method with 3 h of magnetic stirring and 6 h of ultra-sonification process at constant mass fraction percentage which was 0.10%. The SEM model used for this study was Tescan Clara which has the magnification capability from 2 to 2,000,000× with scanning mode ranging from 5 to 10,000 microns in width.

The two pure nanoparticles of ZnO and CuO are observed in Figure 3a,e, respectively. From the two samples, distinct differences of two nanoparticles are noticeable with pure ZnO observed to have a flat and wide surface morphology and a petal-like structure when viewed under 50.0 k× magnification, while the CuO nanoparticle has a round spherical surface morphology when viewed under similar magnification of 50.0 k×. Figure 3b–d are the SEM images of hybrid nanofluids dispersed in water mixed at different ratios. When the ZnO concentration is higher than CuO (at ratio 7:3 of ZnO-CuO), more petal-like structure can be observed and the same can be said vice versa when CuO concentration is higher than ZuO (at 3:7 ratio). It is observed that the gaps between the hybrid nanoparticles at ratio 5:5 are significantly lesser in comparison to other mixing ratios as observed in Figure 3c. The spaces between ZnO particles are filled by the spherical structure of the CuO nanoparticles. This effectively improved the collision rate between particles as well as increasing the specific surface area of the nanoparticles in the mixture. The combination of the ZnO and CuO nanoparticles significantly enhanced the specific surface area of the particles as well as the collisions number between the two nanoparticles when mixed in DI water. This in turn led to the enhancement of heat transfer properties of the hybrid nanofluid. Considering these factors, the ratio of 5:5 of the ZnO-CuO/water hybrid nanofluid was chosen as the ratio of interest for the jet impingement working fluid for this experiment. To further verify the metal component and the distribution of the hybrid fluids in the mixture, EDX of the component based on SEM is necessary to have a better overall view of the hybrid nanofluids that are mixed at different ratio.

#### 2.2.2. Energy Dispersive X-ray Analysis (EDX)

**Figure 4:**
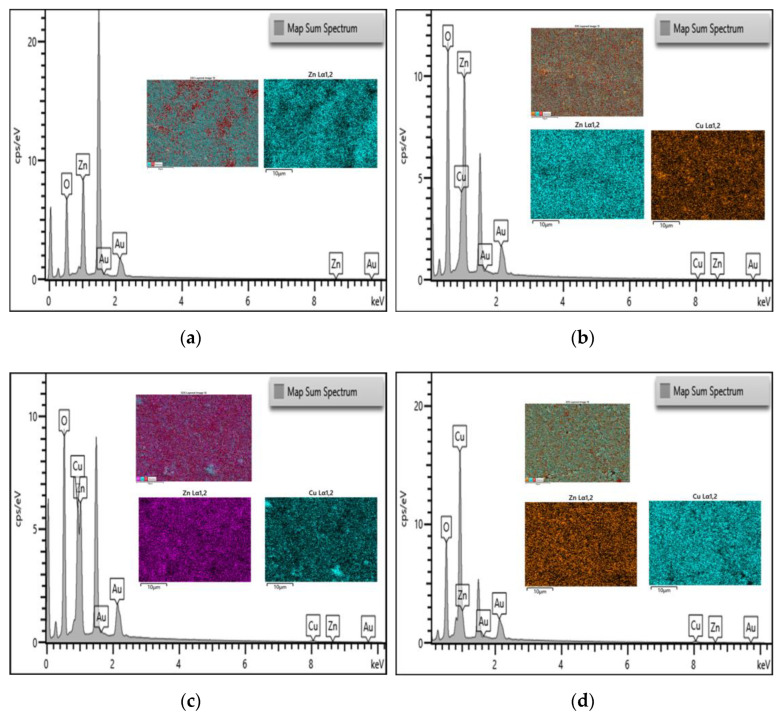

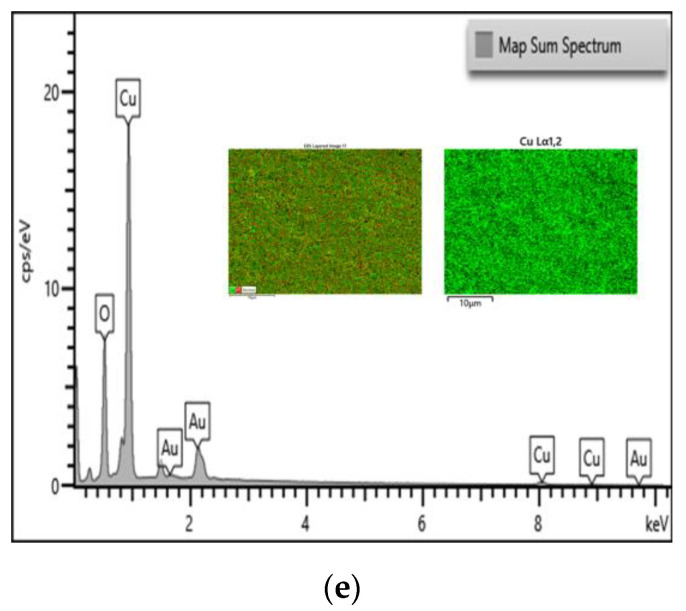
EDX quantitative spectra chart results of ZnO-CuO/water hybrid nanofluid with pattern mapping of the element distribution mixed at ratio of (**a**) 10:0, (**b**) 7:3, (**c**) 5:5, (**d**) 3:7, (**e**) 0:10.

Figure 4 shows the EDX mapping pattern of the element distribution, and quantitative spectra of the five different ratios tested. A larger concentrated element is represented by higher peak in the X-ray energy spectrum. The peaks show the presence of respective elements at different ratio. For all the five samples subjected to EDX, common elements such as gold (Au) and oxygen (O) were found. The figure shows that the EDX results only detected investigated elements in the samples, hence indicating that the preparation process did not contain any impurity or contamination. The EDX results of mono-nanofluids of ZnO and CuO nanofluids are shown Figure 4a,e, respectively. The results confirm that the SEM images of the ZnO and CuO nanofluids reflect the element and the morphological structure observed is ZnO and CuO nanoparticles as mentioned in the previous section. At a mixing ratio of 5:5, Zn and Cu elements were detected with comparable peaks. This indicates that the element was well dispersed during the preparation method. The Au (gold) element detected represents the coating element for the EDX analysis which can be seen having a constant peak intensity for all the five samples. The presence of carbon and oxygen can also be detected across all five samples. The high percentage of O_2_ (oxygen) element detected is due to the nanoparticles from the metal oxide group which have an O functional group, thus reflecting peak for oxygen.

### 2.3. Experimental Setup and Procedure

**Figure 5:**
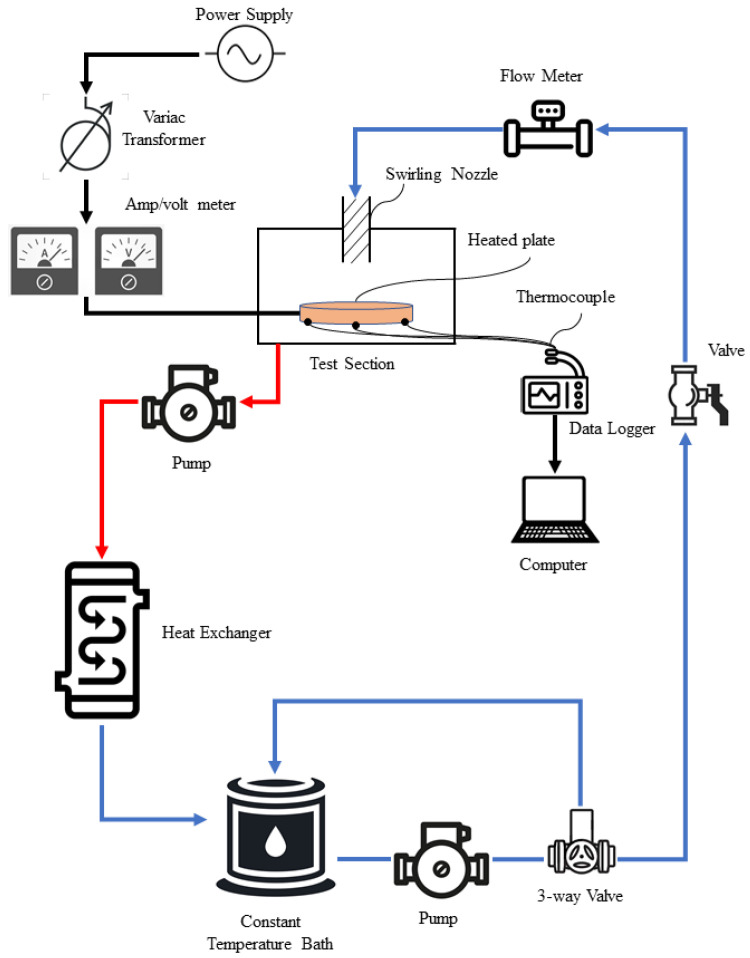
Schematic diagram of the experiment setup.

**Figure 6:**
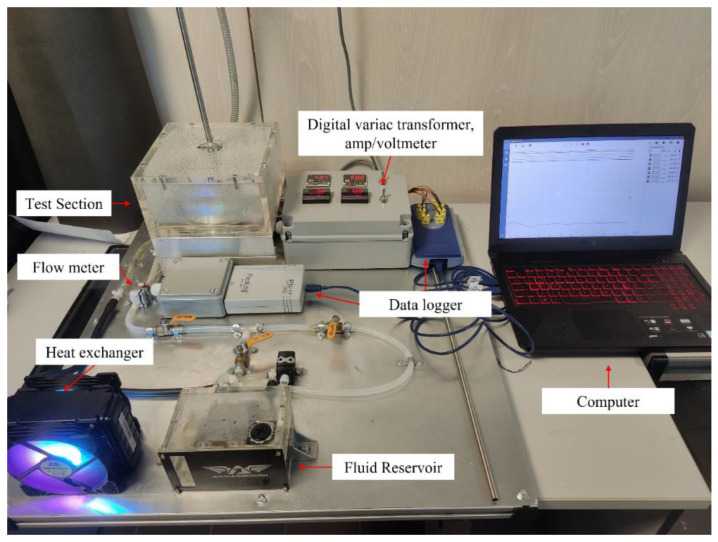
Photograph of experimental setup.

**Figure 7:**
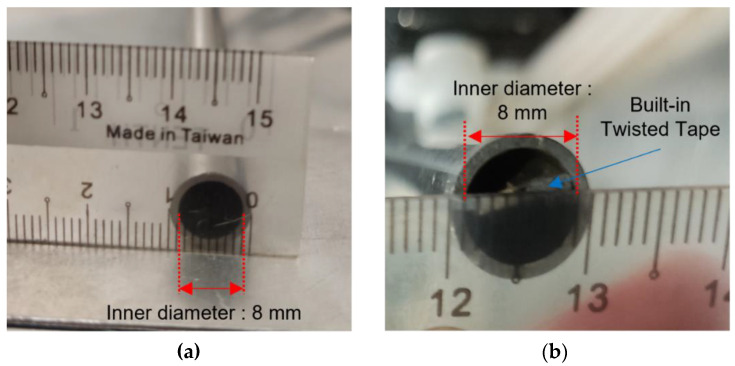
Type of jet impinging nozzles. (**a**) Conventional nozzle, (**b**) twisted tape nozzle.

The experiment setup mainly consisted of heat exchanger with a storage reservoir, a data collection system, and test section for jet impinging. The overall schematic diagram of the experiment setup is shown in Figure 5 while the photograph of the actual experimental setup for this study is shown in Figure 6. The heat exchanger system consists of a cooling fan and a 1 liter storage reservoir tank. For data collection purposes, the system comprises of flow meter, control valve, variac transformer, digital amp/voltmeter, thermocouples, data logger, and a computer. The closed loop test system consists of a jet nozzle and an impinged circular plate (radius of 15 cm and 5 mm thickness) that is heated at constant flux. A conventional nozzle and swirl generator nozzle were used in this study. Both types of nozzles have the same outer diameter of 10 mm and an inner diameter of 8 mm. The swirl generator nozzle was inserted with an aluminum twisted tape (8 mm length) to create the swirl flow effect as shown in Figure 7. The impinged target plate consists of thermocouple (K-type) to read the temperature of the impinged plate through a computer. 

The experiment was conducted in an environmental room temperature of about 25 °C. From the schematic diagram shown in Figure 5, the flow of the cooling system initiated from the fluid reservoir tank (constant temperature bath). A 3-way valve was installed to remove excessive flow of the cooling fluids as well as to have a wider control on the fluid flow. Constant temperature of the cooling fluids from the reservoir pumped and flow into the flow meter as indicated by the blue arrows, before being impinged onto the heated plated. After the working fluids cooled the target surface, the working fluid was collected in the fluid collection tank and passed through the heat exchanger to release the heat absorbed. The heated fluids which are indicated by the red arrows were then pumped to a heat exchanger system to remove the heat gained from cooling the heated plate and flowed back into the reservoir tank. The cooling system was installed with a flow meter to measure the volumetric flow rate of the working fluid. The inlet temperature was measured as T_i_ and was kept constant throughout the experiment. Before the cooling process where the fluid accumulated in the fluid reservoir tank, the working fluid temperature T_o_ was measured. The upper surface temperature, T_us_ (impinged surface), was then obtained using a thermographer imager as well as seven thermocouples which were installed below the heated plate.

### 2.4. Data Processing and Error Analysis

Before any data collection of the experiment, the jet impingement system was run continuously for 5 min before data collection process, in order to ensure the system achieved steady flow condition.

The average temperature of hybrid nanofluids can be calculated as follows:

(1) $$\bar{T}=\frac{T_{\mathrm{in}}+T_{\mathrm{out}}}{2}$$

T_in_ is the temperature of the working fluids before being impinged on the heated plate while T_out_ indicates the temperature outlet of the working fluids after they absorbed the heat from the heated plate. Input heat, q, on the stainless-steel heater pad is obtained through the equation as follows:

(2) $$q\;=\frac{P}{A}$$

P is the power input which was obtained through the digital display of the data logger. The heat exchange from the impinged surface to the thermocouple underneath the heated plate may cause variation in data collection. The additional distance between heated plate surface to the thermocouples may induced errors in temperature measurement which will lead to nonlinear change in error coefficient. Thus, to mitigate the experimental error during data collection phase, thermal resistance of the stainless-steel impinged surface to the temperature measuring point of thermocouples can be calculated with the following equations [29,30]:

(3) $$T_{s}={T_{s}}^{\prime }-\frac{q\delta }{k}$$

(4) $$\Delta T=T_{s}-\bar{T}$$

From the Equations, T_s_ is the temperature of the impinged surface while T_s_′ is the temperature of the thermocouples measured in the stainless-steel heated plate. δ in this study is the distance from the impinged surface to the temperature measurement point in the heated plate. The density of the hybrid nanoparticles is obtained by using empirical formula as follows [31,32,33,34]:

(5) $$\left[\left(1-\phi_{2}\right)\left\{\left(1-\phi_{1}\right)\rho_{f}+\phi_{1}\rho_{s1}\right\}\right]+\phi_{2}\rho_{s2}$$

The coefficient of heat transfer is defined as:

(6) $$h=\frac{q}{A\cdot \Delta T}$$

Reynolds number and Nusselt number of the working fluids is then calculated with the following equation:

(7) Re=ρVDμ

(8) $$\mathrm{Nu}=\frac{\mathrm{hD}}{k}$$

Table 1 shows the thermophysical properties of metal-oxide-based nanoparticles ZnO and CuO together with water as base fluid at 25 °C. It is expected that the hybrid nanofluid would have a higher Nusselt number due to the dispersed nanoparticle which have a higher thermal conductivity when compared to base fluid. Details of each component used in the jet impingement system is shown in Table 2 together with the component error ranges. Furthermore, the error in measurement of the system is shown in Table 3. In addition, Table 4 provides the uncertainty value and equation of the jet impingement cooling experiment.

## 3. Results and Discussion

### 3.1. Stability of Hybrid Nanofluids

**Figure 8:**
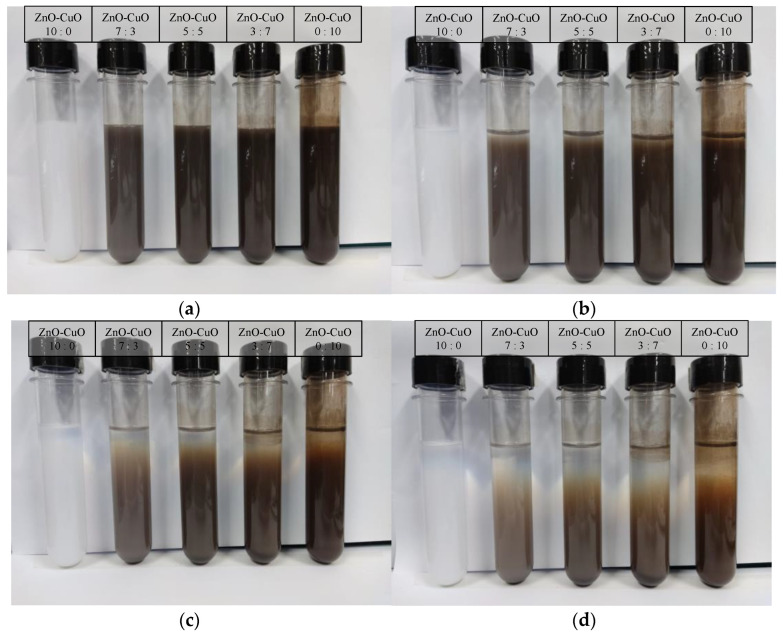
Visual observation on the sedimentation for different mixing ratio of ZnO-CuO/water hybrid nanofluids without surfactant-SDBS at (**a**) 24 h, (**b**) 48 h, (**c**) 72 h, and (**d**) 96 h.

**Figure 9:**
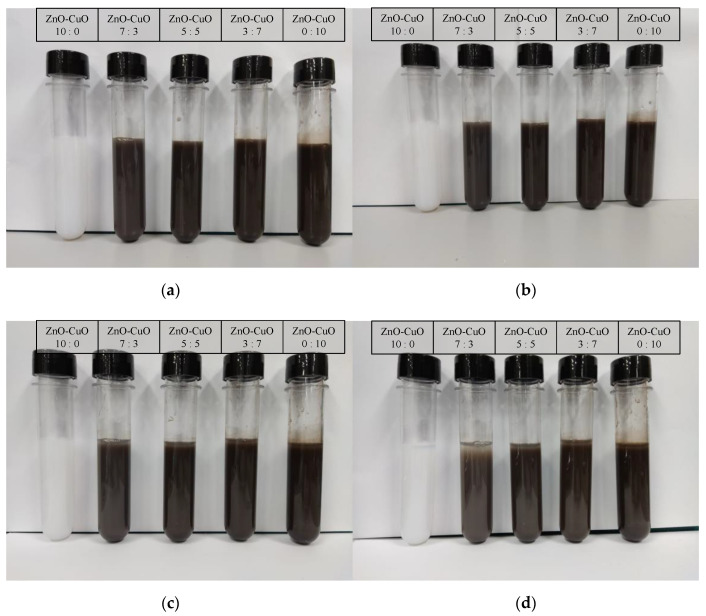
Visual observation on the sedimentation for different mixing ratio of ZnO-CuO/water hybrid nanofluids with surfactant-SDBS at (**a**) 24 h, (**b**) 48 h, (**c**) 72 h, and (**d**) 96 h.

The dispersants of the hybrid nanofluids prepared were analyzed by adding surfactant-sodium dodecyl benzene sulfonate (SDBS). This surfactant was chosen as it is the most commonly used dispersant by researchers and is reported to positively impact the stability of hybrid nanofluids. Visual observation of the sedimentation of nanoparticles as well as zeta potential study was used to determine the stability of the prepared hybrid nanofluids. Figure 8 shows the results of ZnO-CuO/water hybrid nanofluids mixed at different proportions and staged in a test tube for 4 days. It was observed that the sedimentation began to take place at day 2 where the color of the mixture gradually changed over time. On the other hand, another set of mixture was prepared through similar method but with added SDBS as shown in Figure 9. The added mixture was observed to have a higher stability. Hence, without SDBS added, the hybrid nanofluids are unstable and settlement occurs at a faster rate.

**Figure 10:**
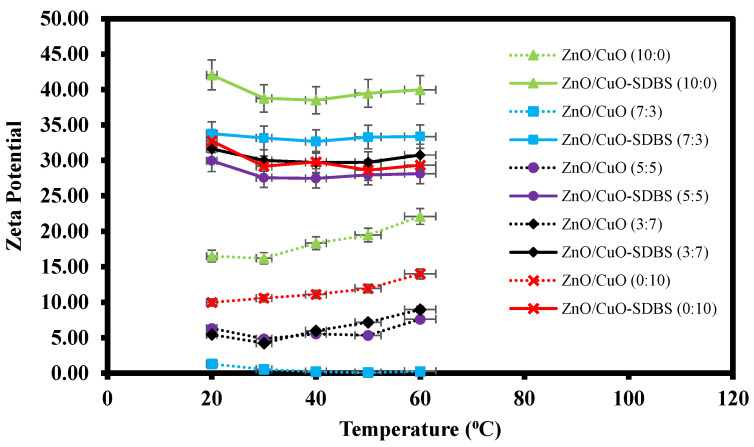
Zeta potential measurement of ZnO-CuO/water hybrid nanofluids at different mixing ratios.

**Figure 11:**
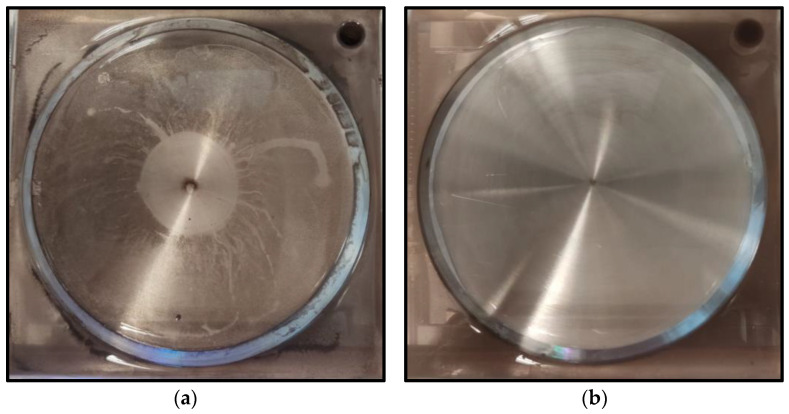
Heated plate surface after being jet impinged (**a**) without SDBS added to hybrid nanofluids; (**b**) with SDBS added to hybrid nanofluids.

A similar set of samples was tested for zeta potential measurement, a technique for detecting the surface charge of nanoparticles in a colloidal solution is often used by researchers to optimize the formulation of suspensions, emulsions, and dispersions by providing precise insight into the causes of flocculation, aggregation, or dispersion. By definition, zeta potential is the voltage at the slipping plane or also known as the edge of diffuse layer where the nanoparticles meet the surrounding base fluids. Instability of the hybrid nanofluids occurs when the repulsive forces surrounding the nanoparticles dropped below a certain value, where the nanoparticles begin to conjugate and slowly sediment. Figure 10 shows the result of zeta potential measurement for hybrid nanofluids with and without SDBS. Hybrid nanofluids with added SDBS were shown to have higher zeta potential value and were considered to be more stable as compared to hybrid nanofluids prepared without surfactant. Lower zeta potential value tended to agglomerate at a faster rate and can be considered as unstable suspension. The surface charge of the nanoparticles was retained due to the adsorption of SDBS which acts as ionic surfactant. This created a counter balance Van der Waal force of attraction and provided electrostatic repulsive force acting on two similarly charged nanoparticles [32]. In short, the results show that the presence of SDBS plays a significant role in enhancing the stability of the hybrid nanofluids. The impact of SDBS on hybrid nanofluids stability can also be seen when used in jet impinging systems as shown in Figure 11. The presence of SDBS aids in a proper dispersion process and helps increase the stability and thus sedimentation of nanoparticles is not observed on the surface of the heated plate.

### 3.2. Thermal Conductivity and Viscosity of Hybrid Nanofluids

**Figure 12:**
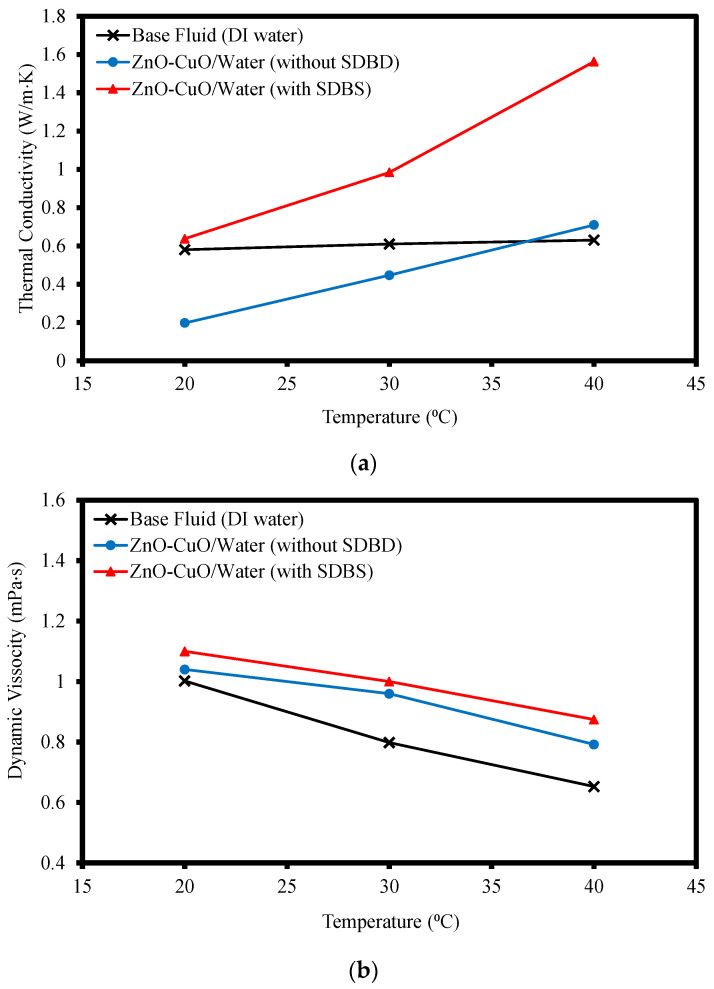
(**a**) Thermal conductivity and (**b**) viscosity of ZnO-CuO/water hybrid nanofluids at 5:5 ratio and DI water at temperature ranging 20–40 °C.

Figure 12 shows the thermal conductivity and viscosity of ZnO-CuO/water hybrid nanofluid with and without the mixture of surfactant-SDBS as well as water as a mean of comparison to the baseline. All three samples were tested at temperatures ranging from 20 to 40 °C. From Figure 12a, the hybrid nanofluid mixed with surfactant has the highest thermal conductivity as compared to the base fluid and the hybrid nanofluid without the present of surfactant. This shows that the presence of nanoparticles promoted micro-convection in the fluid which in turn enhanced the heat transfer rate [33]. The stability of the hybrid nanofluids is crucial in ensuring the heat received is well dispersed among the fluids for a better convection rate. Instability of the hybrid nanofluids due to agglomeration shows that the hybrid nanofluids performed worse than the base fluid from temperatures 20–30 °C, but as temperature rose to 40 °C, the presence of metal-oxide nanoparticles aided in improving the heat transfer rate as compared to water but still under-performed as compared to stable hybrid nanofluids with surfactant SDBS. In this study, high thermal conductivity value is important as it carries a better ability to disperse heat at a better rate and thus improves the jet impinging cooling performance. Alternatively, the viscosity of the hybrid nanofluid and water were observed to be in a decreasing behavior as temperature raises as shown in Figure 12b. This is because the higher temperature led to higher thermal energy gained in the fluids, which accelerated the particles and caused them to become more capable of breaking the attractive forces that bind it together. The addition of nanoparticles and SDBS increased the molecular interaction with the base fluid. Hence, it was observed that the hybrid nanofluid with SDBS added hade a slightly higher viscosity value than hybrid nanofluid without the surfactant added, while water had the lowest viscosity.

### 3.3. Experimental Results and Analysis

#### 3.3.1. Validation Test of Experimental Approach

**Figure 13:**
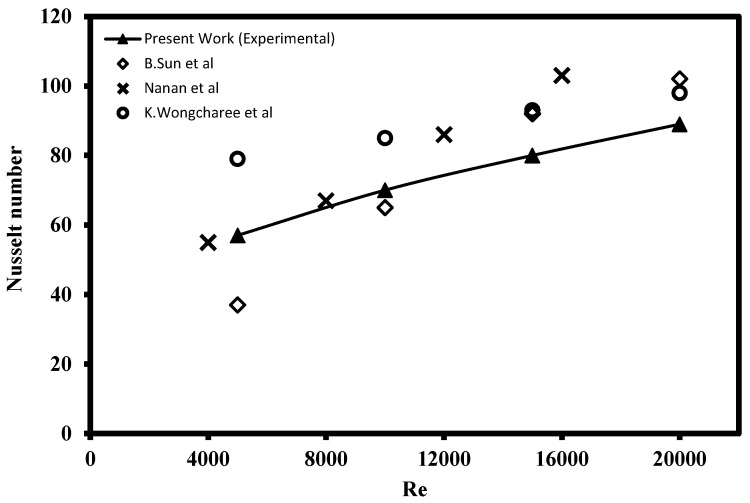
Nusselt number validation test of the experimental swirling impinging jet with water at H/D = 4.

The Nusselt number over the heated plate data from the experimental jet impinging system was collected and a verification test was performed by comparing the outcomes of the CIJ with those reported by Sun et al. [13], Nanan et al. [34], and Wongcharee et al. [35] under similar operating conditions. The validation test was conducted with Re ranging from 5000 to 20,000 using DI water as the cooling agent. The results of the comparison indicate the present jet impinging system is rationally accurate and comparable to the past published works as shown in Figure 13.

#### 3.3.2. Thermal Performance of Jet Impingement

**Figure 14:**
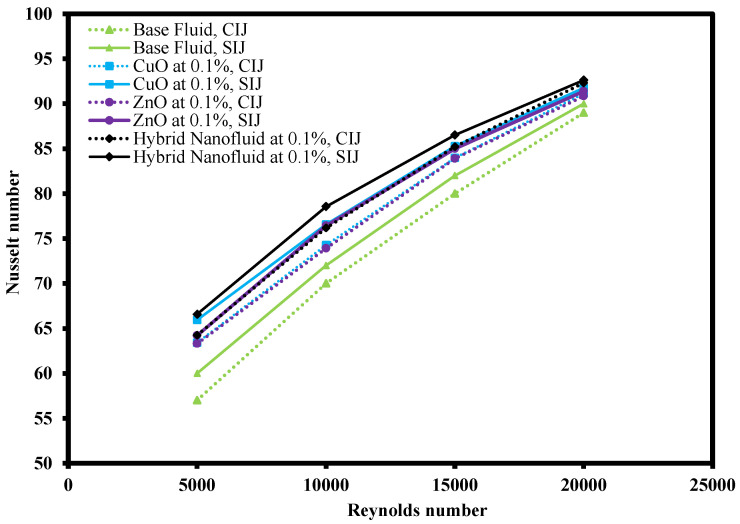
Result of Nusselt number vs. Reynold number for hybrid nanofluid, mono-nanofluids, and base fluids under CIJ and SIJ.

**Figure 15:**
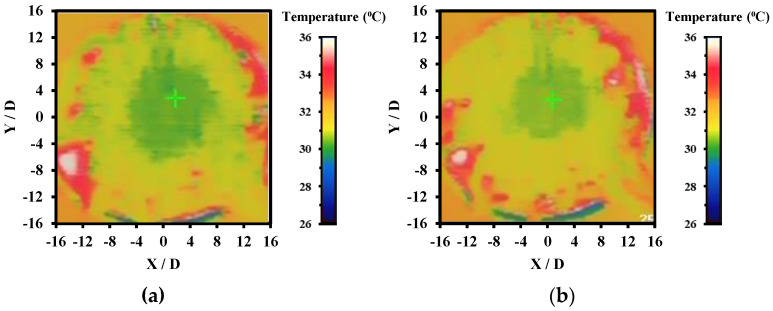
Thermographer images of (**a**) SIJ and (**b**) CIJ on heated plate.

Different types of working fluids operating under conventional impinging jet (CIJ) and swirling impinging jet (SIJ) were compared as shown in Figure 14. The working fluids used were base fluid, CuO-water nanofluid, ZnO-water nanofluid, and ZnO-CuO/water hybrid nanofluid. All the working fluids were subjected to similar experimental conditions, namely Re ranging from 5000 to 20,000 with jet-to-plate distance ratio at 4. From the comparison, the Nusselt number of all the working fluids increased with the increase of Re number in both CIJ and SIJ. Hence, this implies that the heat transfer coefficient of the working fluids is affected by the rate of fluids flow. At an elevated Re number situation, the jet impinged onto the heated plate with higher velocity and there was higher turbulence flow on the heated plate which was able to dissipate the heat faster. From the figure, it was observed that ZnO-CuO/water hybrid nanofluid in SIJ had the highest Nu number compared to base fluids and mono-nanofluids at the same mass fraction and jet-to-plate distance ratio. In general, it was also observed that SIJ offered better heat transfer characteristics as compared to CIJ. This is because the swirling effect of the jet increased the impinged area on the heated plate and increased the overall heat transfer rate on the heated plate. Figure 15 shows the thermography images of the heated plate when subjected to SIJ and CIJ, which demonstrates that under similar experimental conditions, the SIJ had a wider impinged area while CIJ had a localized impinged area. This is due to the built-in twisted tape inserted in the SIJ which entrains more hybrid nanofluids to cover more surface area on the heated plate as compared to CIJ during the jet impingement cooling process. Due to larger area being impinged with hybrid nanofluids, the convection rate and the Nusselt number were higher as heat from the heated plate managed to be dissipated faster.

#### 3.3.3. Effect of Mass Fraction

**Figure 16:**
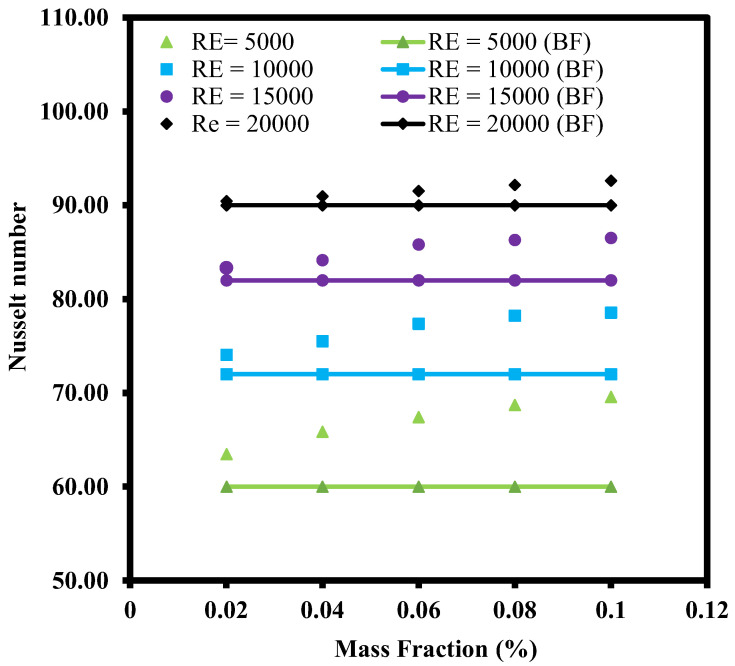
Effect of hybrid nanofluid mass fraction on Nusselt number for SIJ at H/D = 4.

Figure 16 shows the changes of Nu with Re number for ZnO-CuO/water hybrid nanofluids in SIJ with mass fraction ranging from 0.02% to 0.10% at H/D = 4. DI water as base fluid was used as a reference case for comparison purpose. From the chart, it could also be observed that the Nu number increased with the Re number. This is because at a lower Re number, the fluid flow was slower and had less turbulence as compared to fluids at a higher Re number. However, at similar Re numbers, the data showed that Nu number over the heated plate increased with the increase of mass fraction. The higher the amount of hybrid nanoparticles added to the base fluid, the higher the mass fraction percentage which followed by the higher the thermal conductivity or lower the thermal resistance of the hybrid nanofluid. For all the mass fraction values investigated, it was observed that even at low mass fraction value, the heat transfer was better in hybrid nanofluids as compared to base fluids. This is due to the enhanced convection provided by the higher thermal conductivities in hybrid nanofluid as compared to DI water. The presence of nanoparticles in the base fluid also enhanced the heat transfer as the ZnO and CuO nanoparticles were capable of absorbing the heat from the heated plate and dissipate the heat as it flowed through the heat exchanger. The best thermal conductivity enhancement observed was at the highest mass fraction of 0.10%. At an elevated amount of concentration, the rate of collision between particles also increased which eventually led to higher heat transfer coefficient.

**Figure 17:**
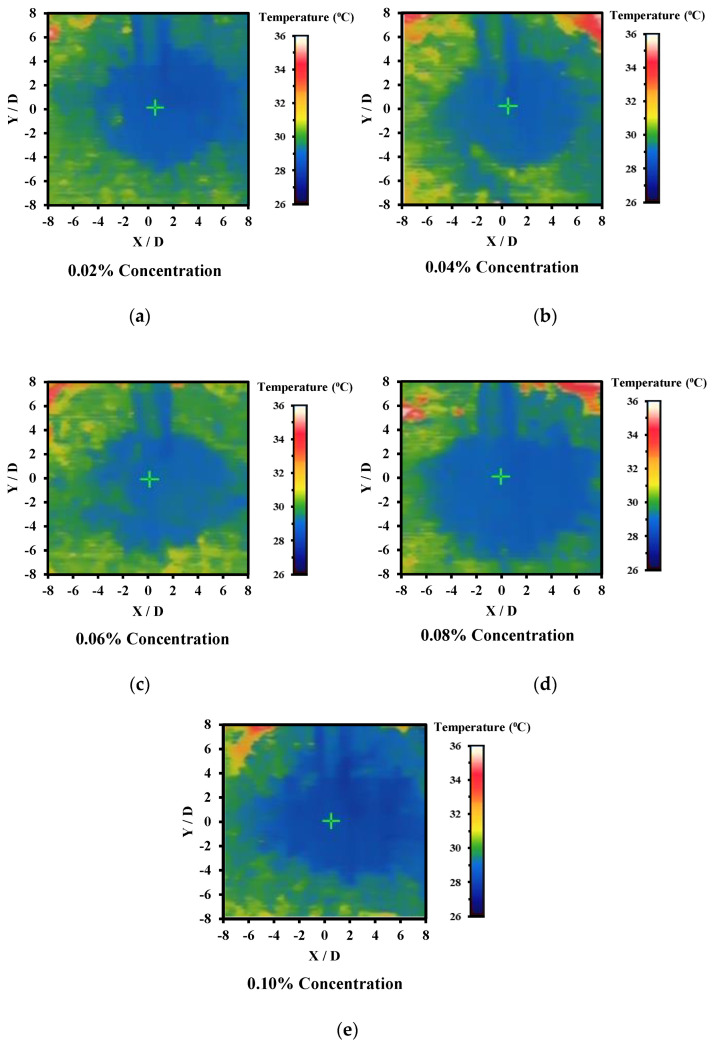
Thermography images of heated plate focusing on stagnation zone of the impinging jet by using hybrid nanofluids at mass fraction (**a**) 0.02%, (**b**) 0.04%, (**c**) 0.06%, (**d**) 0.08%, and (**e**) 0.10%.

From the thermography images as demonstrated in Figure 17, the highest cooling zone was the stagnation zone where the area exhibited is blue in color which reflects lower temperature as compared to surrounding region in green, yellow, or red. The temperature indicator is also attached in each of the thermographer images as illustrated in Figure 17. The stagnation region also indicated high Nusselt number as the rate of heat transfer was the highest at that region where the nozzle jet outlet impinged onto the heated plate. Intensity of the blue region was also observed to be in an increasing trend following the increase of mass fraction. As the concentration of the hybrid nanofluid increased, the thermal conductivity of the fluid increased as well as the viscosity of the fluid. Consequently, it led to enhancement in Brownian motion of the ZnO and CuO nanoparticles in DI water which promoted the thermal diffusion effect. Therefore, more heat was extracted from the heated plate surface as the concentration increased through forced convection.

#### 3.3.4. Effect of Various H/D Values

**Figure 18:**
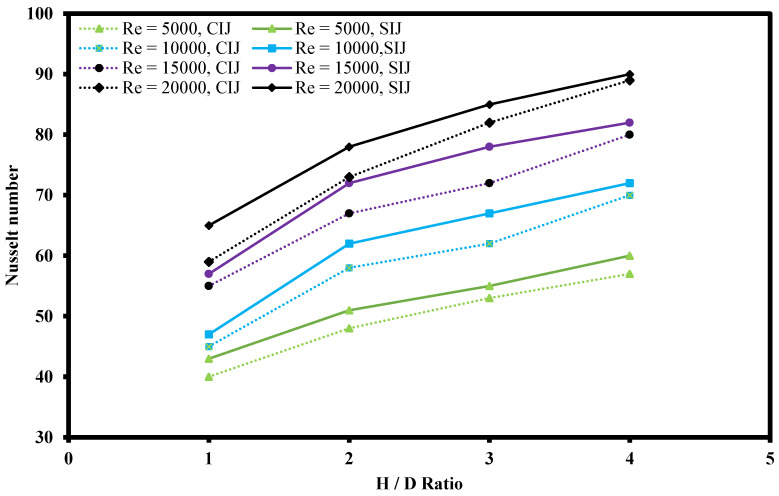
Effect of H/D ratio on Nusselt number for SIJ and CIJ.

**Figure 19:**
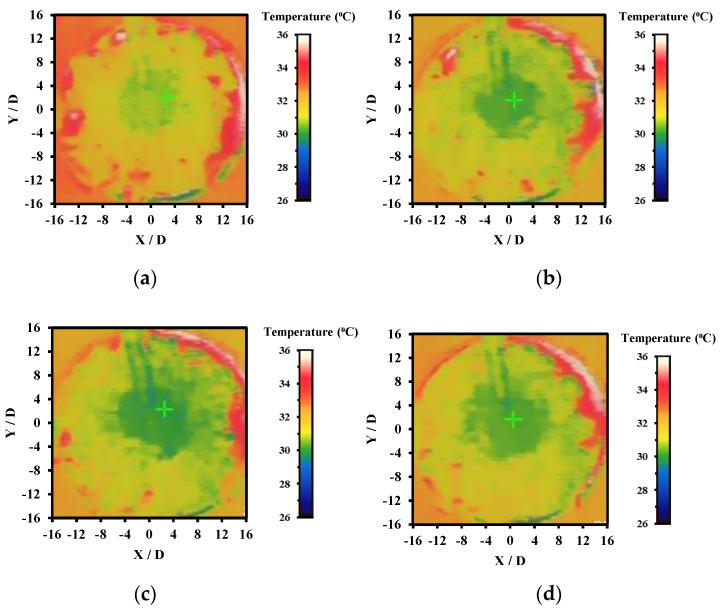
Thermography images of SIJ at (**a**) H/D = 1, (**b**) H/D = 2. (**c**) H/D = 3, and (**d**) H/D = 4.

Figure 18 shows the effect of jet-to-plate ratio (H/D) ranging from 1 to 4 over the Nusselt number on heated plate with constant mass fraction at 0.10% of hybrid nanofluid. It was clearly observed that with increasing Re numbers, the Nusselt number increased as well at all H/D ratios. This is because higher turbulence flow of hybrid nanofluid increases the convection heat transfer coefficient as well as promoting mixing of the fluid. This, in turn, enhances momentum, friction force, and heat transfer between the working fluids and the heated plate. Another critical piece of information that can be extracted from Figure 18 is that, at constant Re number, with increasing H/D ratio, the Nusselt number tended to increase in both the CIJ and the SIJ system. This is because at lower H/D ratio, the nozzle jet outlet was very close to the heated plate, thus leading to the hybrid nanofluid outlet flow being impeded and restricted, limiting the coverage of the fluid flow over the heated plate. However, when the H/D ratio increased, the jet impinged area also increased where the convection rate was also better as shown with thermography images in Figure 19. The surrounding hot spot temperature as indicated in red color at the edge of the heated plate was observed to be decreased as the H/D ratio increased, thus indicating that the impinged area was larger at higher H/D ratios. This is because the nozzle tip of the jet impinging system was not restricted and the impinged area managed to spread over a larger area on the heated plate. In addition, at higher H/D ratios, the intensity of the jet impinged was larger which resulted in an enhancement in heat transfer area of the liquid exchange with the heated plate impacted surface. In comparison to CIJ and SIJ, it was observed that SIJ was superior to CIJ at all H/D ratios. This is because the twisted tape in the SIJ caused a strong swirl that increased the jet impact area, forming a larger stagnation region at the center of the impinged heated plate surface. In addition, the presence of twisted tape in the SIJ promoted turbulence of the hybrid nanofluids prior to jet impinging which induced mixture of particles that led to enhanced collisional contact surface area as compared to CIJ.

## 4. Conclusions and Recommendations

### 4.1. Conclusions

The ZnO-CuO/water hybrid nanofluids were successfully prepared through a 2-step method. The prepared hybrid nanofluids were characterized through SEM showing a distinct morphology structure of ZnO and CuO nanoparticles. The selected mixing ratio for the jet impingement cooling system was 5:5 based on the SEM results and EDX results which confirmed the observed elements through quantitative spectra chart of the mixture. The selected mixing ratio may potentially have the best possible heat transfer enhancement capability due to the observed surface structure which was more compact with lesser gaps in between the hybrid nanoparticles. From the stability study through visual inspection, the hybrid nanofluids were found to be more stable with the presence of SDBD surfactant compared to hybrid nanofluids without SDBD added. This visual observation result was further confirmed with a zeta potential study, showing the same positive result where the hybrid nanofluids remained stable for a longer period of time with the presence of SDBD surfactant. The validation tests of the experimental and numerical approach were both in line with the published works by other researchers. Hence, this indicates that the experimental approach for this study was reliable and comparable to existing journals. The investigated parameters for jet impinging cooling system were the Re number, the H/D ratio, concentration of the ZnO-CuO/water hybrid nanofluid, and the effect of swirl flow towards the Nu number of the heated plate. From the results obtained, it was observed that at the higher Re number, the Nu number increased. This was due to the higher velocity of the fluids which increased the turbulence flow that promoted the heat transfer rate between the hybrid nanofluids and heated plate. On the other hand, the H/D ratio showed that, at higher H/D value, the Nu number was higher. This is likely due to the better coverage over the heated plate where the impinged area was larger, thus increasing the heat transfer area. Furthermore, the higher the mass fraction of the hybrid nanofluids, the higher the Nu number. One of the factors contributing to this effect was likely the increases in the particles collision as well as the Brownian motion of the fluids which enhanced the heat transfer rate. Lastly, the effect of heat transmission by swirling impinging jets was better than that of conventional impinging jets under similar operating conditions.

### 4.2. Recommendations for Future Work

- Hybrid nanofluid preparation method

The typical two-step method of hybrid nanofluids preparation is prone to causing agglomeration of the nanoparticles. Further exploration of the one-step method may reveal more insight into the stability capability of the ZnO-CuO/water hybrid nanofluids. The optimum sonification process to promote stability of the hybrid nanofluids was not studied in detail and only SDBS was used as surfactant to improve the stability. Exploration of other surfactants may provide more information on the stability enhancement of the hybrid nanofluids.

- Jet impingement configuration

The heat transfer enhancement investigation was only conducted on free surface jet in this experiment study. The scope of research may be extended to different configuration such as submerged or confined jet in order to obtain the best configuration for heat transfer performance. In addition, the studied target surface is a flat surface. Similar configuration may have a different impact on different types of target surfaces such as concave or convex heated plates.

- Selection of hybrid nanoparticles

Due to the tight budget for this experimental approach, only two types of nanoparticles were selected, ZnO and CuO nanoparticles. Combination of the metal oxide-based nanoparticles with other subgroups such as metal or carbon-based nanoparticles remains a gap in this experiment study.

## Figures and Tables

**Table 1:** Thermophysical properties of base fluid and nanoparticles.

Parameter	Base Fluid	Nanoparticles	
	Water	ZnO	CuO
k (W m^−1^ k^−1^)	0.613	13	18
ρ (kg m^−3^)	997	5600	6320
C_p_ (J kg^−1^ K^−1^)	4179	495.2	540
Particle size (nm)	-	<50	<50
Colour	-	White to yellow	Black
Specific surface area (m^2^g^−1^)		88.89	99.67

**Table 2:** Components details of jet impingement system.

No.	Component Name	Model	Details	Component Error
1	Shimaden digital controller	SRS11A	Measuring range: 0–800 °C Temperature control range: 0–200 °C	±0.25% of full scale (FS)
2	Murata power solutions’ series AC power meter	ACM20	Display frequency: 0.1 Hz	±1% of FS
3	Submersible pump (inlet)	QR50C	Max flow rate: 240 L/h Power consumption: 6 W	±5% of FS
4	Submersible pump (outlet)	QR50D	Max flow rate: 400 L/h Max power: 10 W	±5% of FS
6	Temperature data logger	Picolog-TC-08	Number of channels: 8 Resolution: 20 bits	±0.2% of FS
7	Flow rate data logger	Picolog-1216	Number of channels: 16 Resolution 12 Bits	±0.5% of FS
8	Flow rate sensor	YF-S401	Range: 60–300 L/h	±2 L/min
9	Heater pad	Maltec-h	Size: D = 15 cm Thickness: 0.1 cm Max temp: 200 °C	±5 °C

**Table 3:** Errors in measurement.

Parameter	Accuracy
Heated plate diameter	1 mm
Width of heated plate	0.02 mm
Jet nozzle diameter	0.02 mm
Temperature	0.1 °C
Working fluid flow rate	2 L/min
Time	0.01 s

**Table 4:** Experiment uncertainty value and equations.

Parameter	Equations	Uncertainty
Heat transfer coefficient	$\partial h={\left[{\left(\frac{\partial h}{\partial \dot{q}}\;d\dot{q}\right)}^{2}+{\left(\frac{\partial h}{\partial T_{s}}\;dT_{s}\right)}^{2}{+\left(\frac{\partial \dot{h}}{\partial T_{f}}\;dT_{f}\right)}^{2}\right]}^{\frac{1}{2}}$	7.80
Reynolds number	$\partial Re={\left[{\left(\frac{\partial Re}{\partial V}\;dV\right)}^{2}+{\left(\frac{\partial Re}{\partial D}\;dD\right)}^{2}+{\left(\frac{\partial Re}{\partial \mu }\;d\mu \right)}^{2}\right]}^{\frac{1}{2}}$	5.50
Nusselt number	$\partial Nu={\left[{\left(\frac{\partial Nu}{\partial h}\;\mathrm{dh}\right)}^{2}+{\left(\frac{\partial Nu}{D}\;dD\right)}^{2}+{\left(\frac{\partial \mathrm{Nu}}{\partial k}\;dk\right)}^{2}\right]}^{\frac{1}{2}}$	8.90

## Data Availability

Data available on request due to restrictions. The data presented in this study are available on request from the corresponding author. The data are not publicly available due to privacy and ethical concerns.
